# 

**Published:** 1888-03-31

**Authors:** 


CONTENTS.

**Lying-In Hospitals** - - - - - 437

**Words of Consolation.** Chapter LXXI.—The Sufferings of Christ - - - - - - 438

**The Nursing Mirror.**—British Nursing Association.—Free Training in Midwifery.—English Hospitals on the Continent - 438

**Questions and Answers** - - - - - 438

**The Doctor's Pulpit.**—The Backbones of the Poor - - 439

**Amusements for Convalescents.**—Palmistry Notes. By a Lady - - - - - - 440

**Aids to Nursing.** By M. M. Basil, M.A., M.B., Author of "The Commoner Accidents to Life and Limb."—Food and Feeding - - - - - - 441

**Turning over New Leaves** - - - - - 442

**Notes and News.**—Proposed Hospital Sunday for India.—The Dental Hospital, Leicester Square.—National Association for Supplying Female Medical Aid to the Women of India, etc. - 444

**Annotations.**—Irish Distressed Ladies.—Vegetarianism.—"Sweating." - - - - - - 446

**Rachel Challice's Vow.** By Lina Nevill, Author of "A Future on Trust," etc. - - - - - 447

**The Present State of Metropolitan Lying-In Hospitals** By Mr. Thomas Ryan, Secretary to St. Mary's Hospital, Paddington - - - - - 449

**Letters from Across the Sea** - - - - - v

**Amusements with Profit for all Ages.**—For Men and Women.—Children's Corner.—Puzzles, etc. - - - v

